# A Field‐Friendly, Non‐Toxic Fixative for Integrated Morphological and Molecular Research in Non‐Model Invertebrates

**DOI:** 10.1002/ece3.73006

**Published:** 2026-02-10

**Authors:** Irene del Olmo, Paula Moreno‐Martín, Patricia Álvarez‐Campos, Aida Verdes

**Affiliations:** ^1^ Departamento de Biología Universidad Autónoma de Madrid Madrid Spain; ^2^ Department of Biodiversity and Evolutionary Biology Museo Nacional de Ciencias Naturales (MNCN‐CSIC) Madrid Spain

**Keywords:** fieldwork, KINFix, non‐model organisms, RNA integrity, RNAlater, sample fixation, sample preservation, SEM, single‐cell RNA sequencing

## Abstract

Formalin, ethanol, and *RNAlater* are the most commonly used fixatives for morphological and molecular studies. Formalin is preferred for preserving tissue morphology, whereas ethanol and *RNAlater* are used to obtain high‐quality nucleic acids for molecular analyses, including emerging ‐omics techniques. Over the past few years, the study of non‐model organisms has gained attention, but the lack of laboratory cultures for many species requires collecting and fixing the animals directly in the field. Very often, just a few specimens are secured, limiting the possibility of using multiple fixatives for parallel analyses. A single fixative that preserves both morphology and molecules while being easy to handle in the field would therefore be highly valuable. KINFix, a non‐toxic alcohol‐based fixative, was developed to preserve histology, proteins, and nucleic acids simultaneously, enabling both morphological and molecular analyses with the same sample. Here, we evaluate the suitability of KINFix for electron microscopy, RNA preservation, and cell dissociation for single‐cell RNA sequencing (scRNA‐seq) experiments, using four invertebrate species from different spiralian phyla. Our results demonstrate that KINFix maintains RNA integrity for over 3 months, similarly to other standard fixatives, but also preserves morphology and cellular integrity even after cell dissociation, suggesting its suitability for scRNA‐seq applications. While fixation conditions may require optimization for different species and tissues, our findings highlight KINFix as a cost‐effective, versatile, and valuable fixative that enables a wide range of morphological and molecular studies in non‐model invertebrates. KINFix is particularly useful for field‐based research where sample availability and preservation logistics are especially challenging.

## Graphical abstract

Formalin, ethanol, and RNAlater are commonly used fixatives for morphological and molecular studies, but each has limitations when specimen availability is low, as is often the case with field‐collected non‐model organisms. Here, we evaluate KINFix, a non‐toxic alcohol‐based fixative, in four invertebrate taxa and show that it effectively preserves tissue morphology, RNA integrity, and cellular structure, enabling electron microscopy, bulk RNA‐seq, cell dissociation, and flow cytometry analyses, showing potential for scRNA‐seq studies. Our results show that KINFix is a versatile and practical fixative, particularly useful for field‐based integrative morphological and molecular research.

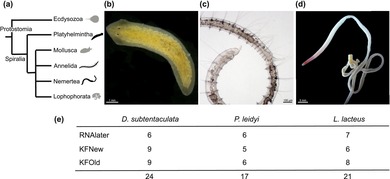

## 1 Introduction

One of the main challenges of working with non‐model organisms in evolutionary and developmental research is to obtain enough number of specimens to resolve a given biological question. Different experimental approaches might require large amounts of tissue or the preservation of multiple specimens in different fixatives (i.e., for morphological vs. molecular studies), posing a significant challenge when laboratory cultures are not available for the focal taxa. For these non‐model organisms, the only practical option is often to collect and fix samples directly in the field. This requires a fixative that preserves both morphology and molecules, while also being easy to handle and store under field conditions. Over the years, different fixatives have been developed to preserve biological samples for a variety of purposes, often tailored to the specific organisms or tissues being studied. Formalin has long been considered the gold‐standard in histology and clinical pathology due to its high efficiency in preserving tissue morphology, its affordability, wide availability, and ease of use. As a result, it has remained the fixative of choice for decades in a range of applications, including histopathological analyses, cellular biology, and even taxonomic studies (e.g., Groelz et al. 2013; Stefanits et al. 2016). However, numerous studies have demonstrated that formaldehyde is a human carcinogen, linked to different pulmonary and neural diseases (Kilburn et al. 1985; Cogliano et al. 2005). In addition to its high toxicity, formalin fixation compromises the quality of nucleic acids, leading to DNA and RNA fragmentation and cross‐linking with proteins, hampering downstream molecular analyses (Campos and Gilbert 2012; Salehi and Najafi 2014; Agne et al. 2022).

Therefore, in recent years, there has been a growing interest in safer, more effective, and better‐suited fixatives to preserve not only tissue morphology and cellular architecture, but also high‐quality nucleic acids and proteins for molecular applications (e.g., Moelans, Oostenrijk, et al. 2011; Moelans, ter Hoeve, et al. 2011; Stefanits et al. 2016). One promising alternative is the non‐crosslinking fixative RCL2 (ALPHELYS, Plaisir, France), which has been successfully applied in immunohistochemistry (Bellet et al. 2008; Preusser et al. 2010), clinical pathology (Delfour et al. 2006; Moelans, ter Hoeve, et al. 2011; Masir et al. 2012), and proteomics studies (Undheim et al. 2014, 2015; Bellet et al. 2008), showing comparable results to formalin fixation. As a result, many laboratories modified their routine protocols to incorporate RCL2. However, RCL2 is a commercial product that has been occasionally discontinued, prompting Stefanits et al. (2016) to develop KINFix (Klinisches Institut für Neurologie Fixative), a cost‐effective, easy‐to‐prepare alternative based on the same core components of RCL2. KINFix preserves tissue morphology as effectively as RCL2 and formalin (Stefanits et al. 2016), while also maintaining the integrity of proteins and nucleic acids. Since its publication, this innovative and affordable fixative has been used in a growing number of studies (Stefanits et al. 2016; Chen et al. 2019; Tanaka et al. 2020; Verdes et al. 2022), demonstrating its value for both morphological and molecular research.

Although KINFix has been mainly applied in histology, its ability to preserve tissue architecture suggests that it could also be suitable for other morphological applications, including scanning electron microscopy (SEM). This approach is particularly relevant for taxonomic studies, where external morphological characters are often key diagnostic features required for proper species identification (Álvarez‐Campos and Verdes 2017; Magarlamov et al. 2018; de Salgado Oliveira et al. 2018; Tilic et al. 2022; Moreno‐Martín et al. 2023). Traditional fixatives like formalin, glutaraldehyde, or ethanol (Kawamoto et al. 2005; Caramelo and Ansemil 2012) are widely used and show great value for this field; however, in some species, they can deform or shrink soft tissues, limiting SEM quality, especially when using small or soft‐bodied species. Thus, testing whether KINFix maintains external morphology would offer a safer, non‐toxic, and broadly applicable alternative for high‐resolution morphological studies. On the other hand, while DNA and RNA have been successfully extracted from KINFix‐fixed paraffin‐embedded tissues for PCR applications (Stefanits et al. 2016), their performance has not yet directly compared to RNA*later* (Invitrogen)*—*the current gold standard for nucleic acid preservation (Mutter et al. 2004). KINFix has proven compatible with some advanced–omics techniques such as MALDI mass spectrometry imaging (MALDI‐IMS) and shotgun liquid chromatography tandem mass spectrometry (LC–MS/MS) (Hempel et al. 2022; Verdes et al. 2022); its suitability for other cutting‐edge approaches, such as single‐cell RNA sequencing (scRNA‐seq), remains unexplored. For many small‐sized organisms, scRNA‐seq requires tissue dissociation of many individuals to provide the cellular resolution required for constructing comprehensive cell atlases (e.g., Sebé‐Pedrós et al. 2018; Massri et al. 2021; Álvarez‐Campos et al. 2024), which poses a significant challenge when working with non‐model organisms that are difficult to culture in the lab. Therefore, a fixative that allows temporary preservation of specimens until additional individuals of the same species and developmental stage can be collected would greatly expand the applicability of these advanced molecular techniques.

In this study, we evaluated the efficiency of KINFix as an alternative to ethanol and formalin in morphological studies requiring SEM. To evaluate its suitability for molecular applications, we compared RNA yield and quality from freshwater and marine invertebrate tissues fixed with KINFix versus RNA*later*. We also tested the stability of the KINFix working solution after storage for over 120 days—the limit tested by Stefanits et al. (2016)—to determine whether crystalized sugar precipitation affects its effectiveness. Lastly, we examined whether KINFix preserves cellular integrity well enough to allow effective tissue dissociation, flow cytometry, and cell permeabilization, for its potential application in scRNA‐seq studies. Our results are particularly relevant for research with non‐model invertebrates directly collected in the field, where specimens are often rare or available in limited numbers, making it difficult to conduct multiple types of analyses with the same samples.

## 2 Materials and Methods

**FIGURE 1:**
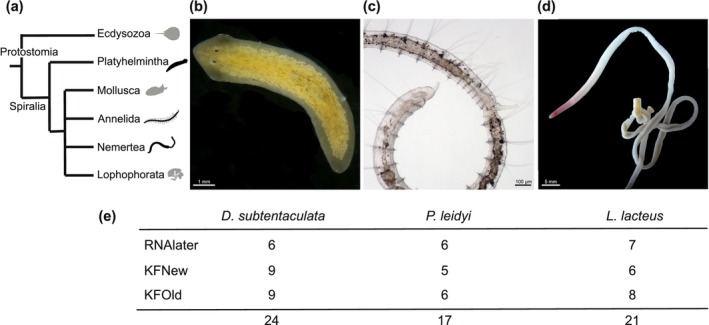
Invertebrate taxa used in this study. (a) Overview of the phylogenetic relationships of the selected species; (b–d) representative specimens of (b) planarian *Dugesia subtentaculata* (Platyhelminthes), (c) annelid *Pristina leidyi* (Annelida), (d) nemertean *Lineus lacteus* (Nemertea); (e) Number of samples analyzed per species and type of fixative used.

We used a set of non‐model spiralian species representing three major soft‐bodied invertebrate phyla—Nemertea, Platyhelminthes, and Annelida (Figure 1). Specimens of the nemertean *Lineus lacteus* , the planarian *Dugesia subtentaculata* , and the annelid *Syllis prolifera* were collected from the field and maintained alive in the laboratory temporarily. We also included the annelid *Pristina leidyi* , which was initially obtained from the company Carolina Biological Supply (North Carolina, United States of America) and used to start a laboratory culture that is maintained through asexual reproduction.

### 2.1 Sample Preparation for Scanning Electron Microscopy Imaging

We used specimens of *P. leidyi—*the most challenging species due to its small size—to test the suitability of KINFix to fix specimens for SEM imaging. Specimens for SEM were fixed following four different protocols: (1) 10% formalin for 10 min, (2) 3% glutaraldehyde buffered with cacodylate for 30 min, (3) KINFix for 30 min, and (4) 5% EtOH for 10 min and then 30% EtOH for another 10 min. Then, all samples were dehydrated through an increasing ethanol series of 50%, 70%, 90%, and 100%, for 10 min each grade. Fixed and dehydrated worms were stored in 100% EtOH at 4°C and then prepared on an Emitech K850 Critical Point Dryer, mounted with adhesive tabs on aluminum stubs, gold‐coated with a Q150T‐S Turbo‐Pumper Sputter Coater, and examined with a Hitachi S‐3000N SEM at the Servicio Interdepartamental de Investigación (SIdI) of the Universidad Autónoma de Madrid (UAM).

### 2.2 Preservation of Specimens for RNA Extraction

We analyzed a total of 62 samples from three of the taxa included in this study (Figure 1): the planarian *D. subtentaculata* (24 samples; Figure 1b), the annelid *P. leidyi* (17 samples; Figure 1c), and the nemertean *L. lacteus* (21 samples; Figure 1d). Between 50 and 100 mg of tissue were used from each sample, which corresponds approximately to one adult planarian, 20 specimens of *P. leidyi* , and a 1 cm body fragment of *L. lacteus* . Samples were preserved using three different treatments (Figure 1e): (1) *RNAlater* solution (Invitrogen) used as control, (2) KINFix solution within 3 months from preparation (KINFix “new,” KFNew), and (3) KINFix solution over 3 months from preparation (KINFix “old,” KFOld). KFOld was used to evaluate the stability of the fixative over time, since Stefanits et al. (2016) only tested the solution up to 3 months after preparation. After fixation with *RNAlater* or KINFix, all samples were incubated overnight at 4°C and then stored at −20°C until RNA extraction was performed. In all cases, the storage time before processing the samples ranged between 8 months and 1 year. The stock solutions of KFNew and KFOld were kept at 4°C as recommended by Stefanits et al. (2016).

### 2.3 Extraction and Quality Assessment of Total RNA

Fixed tissues were introduced in PowerBead Tubes with 1.4 mm ceramic beads (Qiagen) and 1 mL of TRIzol Reagent (Invitrogen) and homogenized using the soft mode (2 cycles of 15 s with 30 s pause at 5800 rpm) in a Precellys Evolution Touch Homogenizer (Bertin Technologies). Total RNA was extracted on ice to avoid RNA degradation, using the phenol‐chloroform method with TRIzol, following the manufacturer's instructions. The quantity and quality of the total RNA extracted were assessed with a NanoDrop 1000 Spectrophotometer (Thermo Fisher Scientific). We measured RNA concentration (ng/μL) and integrity based on absorbance values at 230, 260, and 280 nm. A sample is considered of good quality (i.e., pure) when the A260/280 ratio is ∼2.0, and the A260/230 value is between 2.0 and 2.2 according to the NanoDrop Spectrophotometer manufacturer's instructions. In addition, RNA integrity of three samples per treatment was assessed using the High Sensitivity RNA ScreenTape in the 4150 TapeStation System (Agilent). RNA quality is indicated by the RNA integrity number equivalent (RIN^e^), with values that range from 1 when RNA is degraded to 10 when RNA is intact. RIN^e^ values over 7 are generally considered acceptable for RNA sequencing experiments (Padmanaban et al. 2012).

### 2.4 Statistical Analyses

Differences in RNA concentrations (ng/μL), absorbance ratios (A260/230 and A260/280), and RIN^e^ values were analyzed following the same statistical methods. We first applied the ROUT test to identify and eliminate outliers (*Q* = 1%). We then checked whether the data followed a normal distribution according to the Shapiro–Wilk test (*p* < 0.05). When the data did not follow a normal distribution, a logarithmic transformation was applied, and the Shapiro–Wilk normality test was repeated. To test for significant differences when the data followed a normal distribution, we performed a one‐way ANOVA using Turkey's multiple comparison test (*p* < 0.05). When the data did not follow a normal distribution even after logarithmic transformation, we applied the Kruskal–Wallis non‐parametric test using Dunn's multiple comparison test (*p* < 0.05). This non‐parametric test was also applied to TapeStation RIN^e^ values, as only three measurements per condition were available. All statistical analyses and graphs were performed with the software GraphPad Prism v.9.5 (www.graphpad.com).

### 2.5 Cell Dissociation and Flow Cytometry Analyses

Since tissue architecture and cell structure are well preserved with KINFix, we also wanted to test the possibility of using KINFix‐fixed samples for scRNA‐seq. We used the ACetic‐MEthanol (ACME) dissociation protocol described in García‐Castro et al. (2021) with two different annelid species: the laboratory cultured *P. leidyi* and the field‐collected *S. prolifera* . We used 100 adult individuals of *P. leidyi* and 25 specimens of *S. prolifera* previously fixed in KINFix and preserved at 4°C. As a control, live specimens were directly dissociated using the same ACME protocol. Following dissociation, cells were cryopreserved in DMSO and stored at −80°C for downstream analyses. Thawed samples were filtered through 50‐μm CellTrics strainers (Sysmex) and stained for flow cytometry. Nuclei and cytoplasm were labeled with DRAQ5 and Concanavalin‐A, respectively, and visualized with a CytoFlex S Flow Cytometer (Beckman Coulter) at the SIdI‐UAM. Flow cytometry profiles of KINFix‐fixed, ACME‐dissociated samples were evaluated by identifying distinct gated populations, quantifying the proportion of singlet cells (i.e., DRAQ5‐positive and Concanavalin‐A‐positive single cells) with the staining and gating parameters described in García‐Castro et al. (2021).

Additionally, to assess whether KINFix preserves cell morphology, we compared ACME‐dissociated cells from KINFix‐fixed samples and fresh tissue, labeled with DRAQ5 and Concanavalin‐A to stain nuclei and cytoplasm, respectively, and imaged using a Leica TCS SP5 spectral confocal microscope with a 63× oil immersion objective at the SIdI‐UAM. Images were compiled with Fiji v.2.0 (Wayne Rasband, National Institutes of Health), and final figure plates were assembled in Adobe Illustrator v29.6.1.

## 3 Results

### 3.1 KINFix Effectively Preserves External Morphology for SEM Imaging and Detailed Morphological Assessment

**FIGURE 2:**
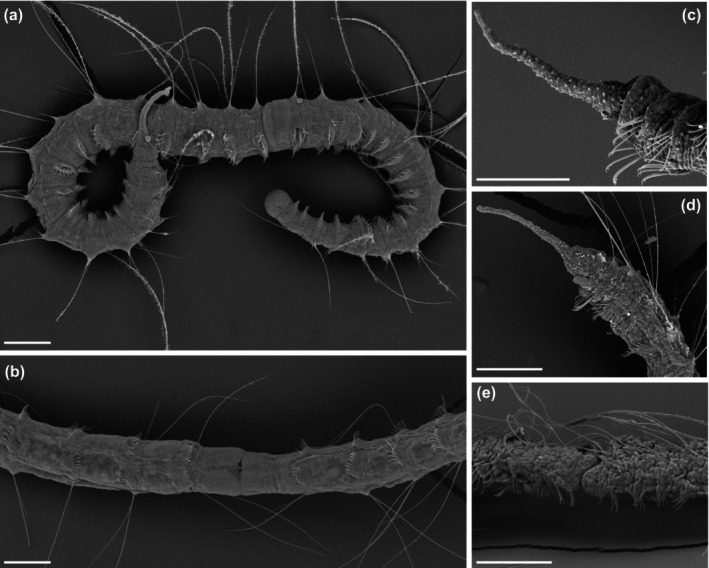
Scanning electron microphotography of *Pristina leidyi* fixed with different solutions. (a) Individual fixed with KINFix, lateral view of the whole worm; (b) Individual fixed with KINFix, detail of the midbody segments; (c) Individual fixed with an ascending ethanol concentration, detail of the anterior part; (d) Individual fixed with 10% formalin, detail of the anterior part; (e) Individual fixed with 3% glutaraldehyde, detail of the midbody segments. All scale bars are 100 μm.

One of the key features of KINFix is its ability to maintain tissue structure, preventing shrinkage during fixation. Here, we compared the quality of external morphology preservation for SEM imaging in *P. leidyi* specimens fixed with three commonly used fixatives for morphological studies in annelids (i.e., formalin, glutaraldehyde, and ethanol), and KINFix (Figure 2). Samples fixed with formalin, glutaraldehyde, and ethanol showed noticeable body shrinkage (Figure 2c–e), hindering the observation of external morphological details. In contrast, specimens fixed with KINFix retained their original body shape and tissue integrity (Figure 2a,b), showing better preservation for detailed morphological analysis with SEM.

### 3.2 Quality and Integrity of RNA Extracted From KINFix‐Fixed Samples Are Compatible With Downstream Molecular Applications

We successfully extracted RNA from all samples fixed with either KINFix or *RNAlater* (Table S1). However, RNA concentration values varied significantly among species, with *L. lacteus* showing the highest concentration, followed by *D. subtentaculata* , and the annelid *P. leidyi* showing significantly lower concentration values (Kruskal–Wallis, *H* = 83.62, *p* < 0.0001).

**FIGURE 3:**
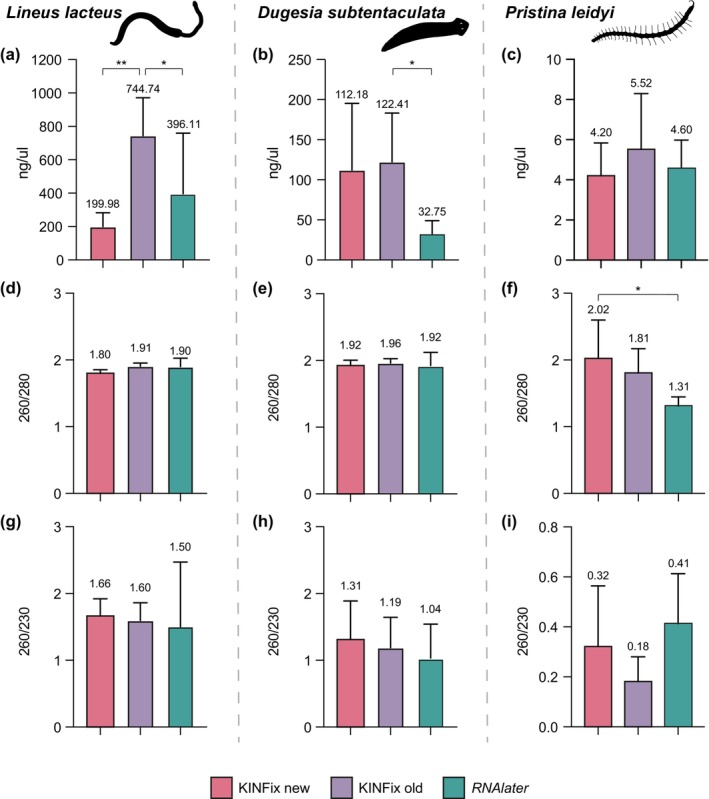
Nanodrop quantification of the total RNA extracted from the samples preserved in KFNew, KFOld, and *RNAlater* for the studied species: *Lineus lacteus* (a, d, g), *Dugesia subtentaculata* (b, e, h), and *Pristina leidyi* (c, f, i); (a–c) RNA concentration; (d–f) 280/260 ratio values; (g–i) 260/230 ratio values. Mean values for each condition are indicated above the bars. Significant differences between samples are indicated by an asterisk (*p ≤ 0.05, **p ≤ 0.01, *p ≤ 0.001, **p ≤ 0.0001).

According to NanoDrop measurements, RNA concentrations were consistently higher in KFOld compared to KFNew and *RNAlater*. In *L. lacteus*, mean values were 744.74 ng/μL for KFOld, 199.98 ng/μL for KFNew, and 396.11 ng/μL for *RNAlater*. In *D. subtentaculata* , values averaged 122.41 ng/μL (KFOld), 112.18 ng/μL (KFNew) and 37.75 ng/μL (*RNAlater*), while *P. leidyi* showed considerably lower mean concentrations 5.52 ng/μL (KFOld), 4.20 ng/μL (KFNew), and 4.60 ng/μL (*RNAlater*) (Figure 3a–c; Table S1). Although *L. lacteus* samples yielded the highest RNA concentrations, ranging from 418.7 to 1014.7 ng/μL with KFOld, and from 114.7 to 315.9 ng/μL with KFnew, differences between KFOld and KFNew were smaller in *D. subtentaculata* (43.5–230.9 vs. 26.2–273.8 ng/μL) and in *P. leidyi* (2.5–9.6 vs. 2.8–6.3 ng/μL). Concentration of samples fixed in RNAlater showed minimal variability in *P. leidyi* (2.9–6 ng/μL), and a broader range in *L. lacteus* (27.35–879.6 ng/μL) and *D. subtentaculata* (12–57.1 ng/μL) (Table S1). Consequently, statistical analysis revealed significant differences in RNA concentration between KFOld and *RNAlater* in the nemertean and planarian samples, but not in the annelid *P. leidyi* (Figure 3a–c; Table 1). No significant differences were found between KFNew and *RNAlater* in any of the three species analyzed (Figure 3a–c; Table 1). In both planarian and nemertean samples, absorbance ratios (260/280 and 260/230) were comparable between KINFix‐ and *RNAlater*‐fixed samples, with similar values across all three conditions (Figure 3d,e,g,h; Table S1). In contrast, *P. leidyi* samples showed a significantly higher 260/280 ratio in KFNew compared to *RNAlater*, indicating slightly better RNA purity, with values closer to 2, in KFNew samples (Figure 3f,i; Table 1; Table S1).

**TABLE 1:** Statistical comparison of the concentration, absorbance and RIN^e^ values measured with the Nanodrop and TapeStation for all samples fixed with KFNew, KFOld and RNAlater.

	Quantity		Integrity		
	Nanodrop	Tapestation	Nanodrop		Tapestation
	Concentration		260/280	260/230	RIN
*Lineus lacteus*	KFOld > KFNew (*p* = 0.0027) KFOld > RNAlater (*p* = 0.0436)	ns*	ns	ns	RNAlater > KFOld (*p* = 0.0338)*
*Dugesia subtentaculata*	KFOld > RNAlater (*p* = 0.0378)	ns*	ns	ns	ns*
*Pristina leidyi*	ns	RNAlater > KFOld (*p* = 0.0338)*	KFNew > RNAlater (*p* = 0.0218)	ns	—

*Note:* Comparisons were performed separately for each species. Only *p*‐values for statistically significant differences (*p* < 0.05) are shown in brackets. Unless otherwise specified, statistical analyses were performed using one‐way ANOVA followed by Tukey's multiple comparisons test. Comparisons marked with an asterisk were analyzed using the Kruskal–Wallis test with Dunn's multiple comparisons, applied when data did not follow a normal distribution.

**FIGURE 4:**
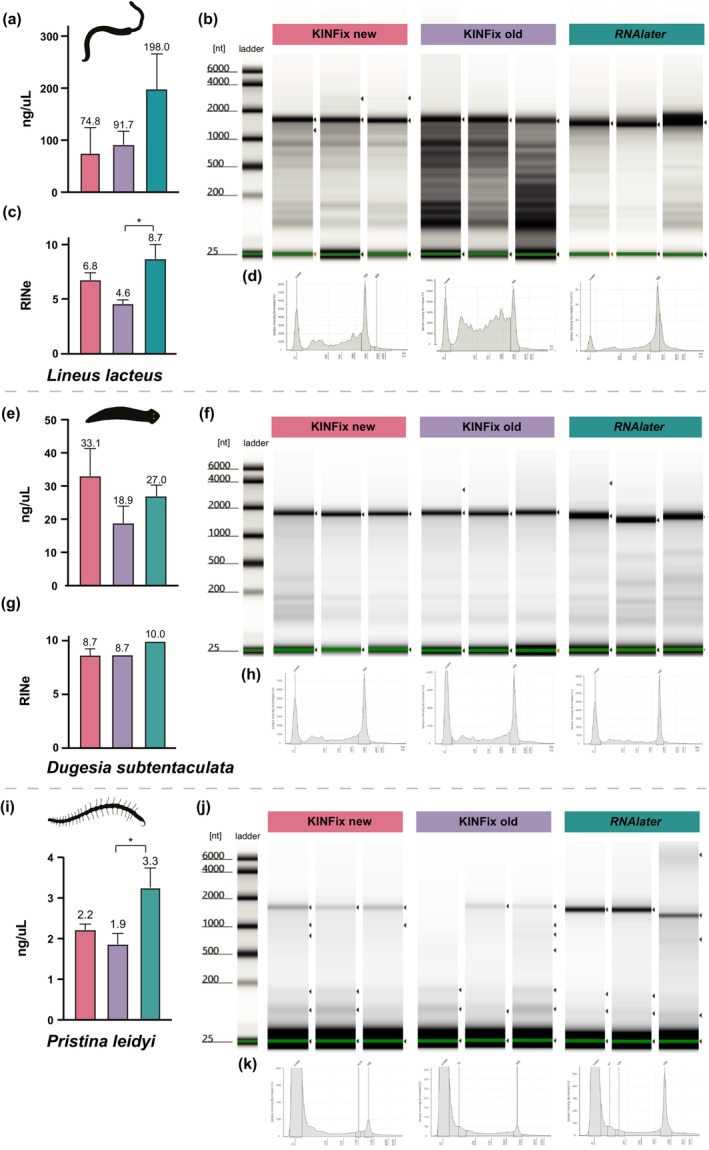
TapeStation profiles of the total RNA extracted from the nemertean *L. lacteus* , the planarian *D. subtentaculata* , and the annelid *P. leidyi* . Three replicates for each condition (KFNew, KFOld, and *RNAlater*) are shown. (a, e, i) Statistical analysis of the total RNA concentration for each condition; (b, f, j) gel images of the different samples analyzed; (c, g) statistical analysis of the RIN^e^ values for each condition. Mean values for each condition are indicated above the bars; (d, h, k) representative electropherogram of total RNA from one replicate per condition. Significant differences between samples are indicated by an asterisk (*p ≤ 0.05).

To validate these results, we further assessed RNA quality using the Agilent TapeStation (Figure 4; Table 1; Table S1). In contrast to Nanodrop results, TapeStation analysis generally showed higher RNA quality of samples fixed in *RNAlater*, especially in *L. lacteus* , where both RNA concentration and RIN^e^ values were significantly higher compared to KINFix‐fixed samples (Figure 4a,c). Samples of both *L. lacteus* and *D. subtentaculata* showed comparable RNA concentrations across all conditions, while significant differences were observed in RIN^e^ values only in *L. lacteus* (Figure 4a,c,e,g). *RNAlater* samples showed slightly higher RIN^e^ values on average than KFNew and KFOld samples (Figure 4e). Only in nemertean samples, significant differences were found between KFOld and RNAlater (Figure 4c; Table 1); however, in planarian samples, RNA integrity was high in all three conditions, with most RIN^e^ values above 8 and a mean of 8.5, except for a single outlier (Figure 4g; Table S1). Electrophenogram profiles were congruent with the significant differences in the lower RIN^e^ values in samples preserved with KFOld, showing highly degraded RNA profiles compared to KFNew and RNAlater (Figure 4b–d). In contrast, electrophenogram profiles revealed the absence of RNA degradation in all KINFix‐preserved planarian samples, demonstrating the fixative's potential to yield high‐quality, non‐degraded RNA (Figure 4f,h). For *P. leidyi* samples, all conditions resulted in very low RNA concentrations, with means of 2.2 for KFNew and 1.9 ng/μL for KFOld, and 3.3 ng/μL for *RNAlater*, with significant differences found only between KFOld and *RNAlater* (Figure 4i; Table S1). Due to these low concentrations outside the quantitative detection range of the TapeStation, RIN^e^ values could not be calculated for these samples (Table S1). Nevertheless, the electrophenogram profiles and gel images show no sign of RNA degradation in any of the three conditions analyzed (Figure 4j,k), confirming the integrity of the extracted RNA despite its low abundance.

### 3.3 Combining KINFix With ACME Allows to Preserve Morphology of Dissociated Cells

**FIGURE 5:**
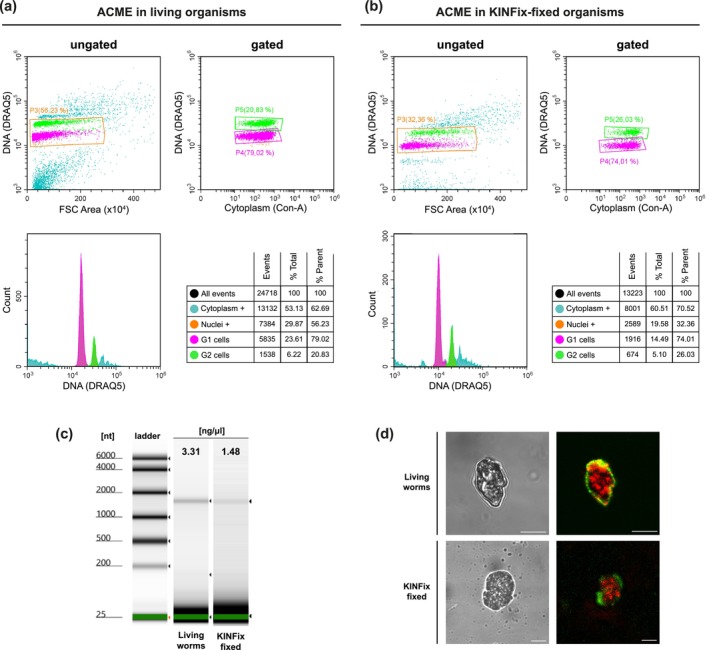
Comparison of ACME‐dissociated cells in live and KINFix‐fixed *Syllis prolifera* individuals. Flow cytometry profiles of ungated and gated profiles of ACME‐fixed cells from (a) live organisms, used as control samples, and (b) KINFix‐fixed samples, stained with DRAQ5 (nucleus) and Concanavalin‐A (cytoplasm). For each comparison (a, b), ungated cytoplasm positive events (blue) and nucleus positive gated population (orange) in the upper‐left corner, gated nucleus‐positive cells resulting in G1 (magenta) and G2 (green) populations in the upper‐right corner, histogram of the DNA content (linear scale) showing the relative proportions of G1 and G2 cells in the bottom‐left corner, table showing the number and percentage of events of the gated populations in the bottom‐right corner. (c) TapeStation profiles and concentration values from total RNA extracted from the ACME dissociations from living organisms and KINFix‐fixed samples. (d) Bright‐field and confocal micrographs from ACME‐dissociated cells from living organisms (top) and KINFix‐fixed organisms (bottom), with nuclei stained in red and cytoplasm in green. All scale bars are 10 μm.

We dissociated *S. prolifera* and *P. leidyi* specimens previously fixed in KINFix and stored at 4°C using the ACME protocol and compared results to those obtained from freshly dissociated live specimens (Figure 5; Figure S1). Our flow cytometry analysis recovered a well‐defined DNA‐containing population of singlet cells (Concanavalin‐ and DRAQ5‐positive cells) in both conditions (Figure 5a,b; Figure S1a,b). These singlets represent two clearly distinguishable cell populations, corresponding to “G1 cells” containing 2c DNA content and “G2 cells” with 4c DNA content. Although the pellet obtained after ACME dissociation was similar in both samples, the total number of cells recovered in KINFix‐fixed samples was lower compared to the dissociations obtained from live animals (Figure 5a,b; Figure S1a,b). For *S. prolifera* dissociations, the analysis of 10 μL of dissociation volume recorded 24,718 cells in fresh tissue versus 13,223 cells in KINFix‐fixed samples. The number of singlet cells gated was also lower (2589 vs. 7384), corresponding to 17.17% and 29.87% of total cells, respectively (Figure 5a,b). Importantly, the relative proportion of total, G1, and G2 populations was comparable between the KINFix‐fixed and live samples (Figure 5a,b). Moreover, we did not find differences in the proportion of cellular debris or aggregates between KINFix‐fixed and live dissociations, and therefore, in both conditions, they could be easily excluded from the singlets (Figure 5a,b). Similar results were observed in cell dissociations performed in *P. leidyi* (Figure S1). RNA extraction from the cell dissociations showed non‐degraded RNA in either living or KINFix‐fixed samples, although RNA concentration was lower compared to live tissue (Figure 5c). This is expected, as a reduced number of cells naturally results in a proportionally lower amount of RNA and aligns with results in other species showing slightly lower RNA concentrations in KINFix‐fixed samples (Figure 4). Importantly, ACME‐dissociated cells from both KINFix‐fixed and live samples exhibited well‐preserved morphology under confocal microscopy (Figure 5d).

## 4 Discussion

Imaging techniques such as light, confocal, and electron microscopy are essential tools for morphological studies, often necessary for species descriptions and taxonomic identifications. Both transmission and scanning electron imaging (TEM and SEM) are commonly used to describe diagnostic features either to identify species or to describe developmental processes in spiralian taxa, including those analyzed in the present study (e.g., Magarlamov et al. 2018; de Salgado Oliveira et al. 2018; Kawamoto et al. 2005). In nemerteans and planarians, external morphological characters are often important for taxonomy; however, histological observations of the internal anatomy usually provide more reliable information for species‐level identifications (e.g., Sundberg et al. 2009; Bartolomaeus and von Döhren 2010; Brubacher et al. 2014; Strand et al. 2014; Winsor and Sluys 2018; de Miguel Bonet and Hartenstein 2024). In contrast, the identification of annelid species often depends on the examination of external traits, such as chaetal morphology, ciliary structures, mating organs, or developmental stages (e.g., Bergter et al. 2004; Yáñez et al. 2006; Caramelo and Ansemil 2012; Álvarez‐Campos and Verdes 2017; Moreno‐Martín et al. 2023).

Preparation of specimens for SEM imaging requires specific fixation and dehydration protocols to ensure complete desiccation before critical point drying. Tissue shrinkage during this process poses a challenge in the preparation of soft‐bodied organisms, particularly annelids. Fixatives like formalin or glutaraldehyde are still commonly used to preserve the morphological features required for taxonomic identifications (e.g., Kawamoto et al. 2005; Caramelo and Ansemil 2012). Fixation in ethanol 96%–98% is also common and can provide good quality SEM images for key diagnostic characters in many groups (e.g., Álvarez‐Campos and Verdes 2017; Tilic et al. 2022; Moreno‐Martín et al. 2023). However, many of these diagnostic characters correspond to hard structures, such as chaetae, whose morphology is not affected by the tissue shrinkage typically induced by ethanol. In contrast, when the aim is to examine the external morphology of soft tissues, ethanol fixation can lead to pronounced deformation. This limitation is especially evident in *Pristina leidyi* due to its reduced body size. In this species, several fixation methods have been developed for the histological analysis of internal structures (Özpolat et al. 2016), but none are optimized for SEM imaging. Moreover, SEM preparation methods commonly used for other freshwater oligochaetes (Bouché et al. 1999; Yáñez et al. 2006) led to complete tissue disintegration or shrinkage in *P. leidyi* (Figure 2). In contrast, our results show that KINFix not only preserves internal histology and morphology, as previously reported (Stefanits et al. 2016), but also represents an effective alternative for high‐quality SEM imaging of the external morphology, even in species with reduced body size like *P. leidyi* .

Transcriptome analysis represents a powerful tool for modern biology by enabling precise quantification of gene expression and providing comprehensive sequence information. However, extracting high‐quality RNA remains challenging due to its rapid degradation, mainly caused by cytoplasmic RNases, making RNA preservation methods essential. Although RNA can be extracted from formalin‐fixed tissues (Evers et al. 2011; Howe et al. 2025), formalin‐induced chemical modifications often compromise RNA integrity and downstream analyses. In transcriptomic applications, such samples typically yield lower‐quality results, with reduced detection efficiency and the potential introduction of analytical biases (Jones et al. 2019). In particular, fixation time is a critical factor, as formalin‐fixation periods exceeding 48 h have a detrimental effect on RNA accessibility and detectability in transcriptomic analyses, resulting in sparser datasets and poorer analytical outcomes (Howe et al. 2025). Thus, *RNAlater* has become a standard solution for preserving tissue samples, as it stabilizes and protects cellular RNA integrity without the need to freeze the tissue (Mutter et al. 2004; Salehi and Najafi 2014). While effective, *RNAlater* is expensive and does not preserve tissue morphology. Other alternatives, such as the nucleic acid preservation (NAP) buffer, can preserve DNA and RNA at a lower cost (Camacho‐Sanchez et al. 2013) but also fail to maintain tissue morphology.

Our results support the use of KINFix as a dual‐purpose fixative to preserve both RNA integrity and tissue morphology. KINFix provides a more affordable solution than *RNAlater* (~7.5 € for 100 mL of KINFix compared to ~189 € for 100 mL of *RNAlater*) with the added benefit of preserving tissue morphology (Stefanits et al. 2016). In addition, by comparing RNA quantity and quality across multiple parameters and methods, we also assessed whether long‐term storage of KINFix (> 120 days) compromises RNA integrity, as precipitation of crystallized sugar may alter the concentration of the solution (Stefanits et al. 2016). In general, RNA concentration and quality were not affected by the fixative used, showing no significant differences in most comparisons (Table 1). Furthermore, when significant differences were detected, RNA extracted from KINFix samples was of sufficient quality for sequencing (Figures 3 and 4; Table 1). Notably, NanoDrop measurements consistently showed higher RNA concentration in KINFix‐fixed samples (Figure 3a,b,f). In *P. leidyi* , the A260/280 ratio—that indicates protein or phenol contamination—was considerably lower in *RNAlater*‐fixed samples than in KINFix‐fixed samples (Figure 3f; Table S1), with none reaching the minimum value of 1.85 expected for pure RNA (Gayral et al. 2011). In addition, absorbance ratios across all species—especially in *P. leidyi* —were generally lower than values indicative of high‐quality RNA, likely due to the very low RNA concentrations (< 10 ng/μL), which can compromise the accuracy of Nanodrop measurements (Table S1).

However, NanoDrop usually overestimates RNA concentration compared to more accurate methods like the TapeStation (Hussing et al. 2015, 2018). Accordingly, our TapeStation results recovered lower RNA concentrations—often at least half—across all samples. This difference was more pronounced in KINFix samples, probably due to ethanol carryover affecting NanoDrop spectrophotometric readings (Oxford Nanopore Technologies documentation, https://nanoporetech.com/documentation). TapeStation results consistently showed higher RNA concentrations and RIN^e^ values in *RNAlater*‐fixed samples, except for *D. subtentaculata* (Figure 4a,c,g,i). Nevertheless, TapeStation measurements show RNA of sufficient quality for sequencing across all samples.

Overall, our results indicate that KINFix is a valuable and cost‐effective alternative for RNA preservation, providing comparable results to the gold standard *RNAlater* with the added advantage of preserving tissue morphology. Moreover, long‐term storage of KINFix does not seem to greatly affect RNA quality, although RNA concentration was lower in samples fixed with KINFix stored for over 120 days, in both the planarian and annelid species (Figure 4e,i). In addition, RNA degradation was considerably higher in *L. lacteus* samples fixed with KFOld (Figure 4d), indicating storage time might impact some species more than others. Therefore, we recommend testing the optimal fixation conditions for each model species and proceeding with caution when using older KINFix preparations.

Single‐cell transcriptomics enables the analysis of thousands of mRNA molecules in large numbers of individual cells. This method has significantly advanced biological research by revealing cellular diversity and dynamic processes that were previously hidden in bulk RNA analyses. Despite its huge potential, several technical challenges remain unsolved, with recent studies focusing on preservation of dissociated cells and avoidance of compositional and gene expression changes during sample processing (e.g., Alles et al. 2017; García‐Castro et al. 2021; Phan et al. 2021; Burja et al. 2022; Gutiérrez‐Franco et al. 2023). However, the need to preserve entire organisms in the field—critical when laboratory culturing is unfeasible or when individuals at the same developmental stage are hard to collect simultaneously—has not been addressed. Our results indicate that KINFix can be effectively combined with the ACME dissociation and fixation protocol to preserve whole organisms, enabling cell dissociation for downstream flow cytometry analyses while preserving both cell morphology and RNA integrity (Figure 5). Although KINFix‐fixed samples yielded lower RNA concentrations as well as fewer recovered cells than fresh tissue (Figure 5; Figure S1), the possibility of preserving whole organisms eliminates the need for immediate processing and allows collection of enough material. While sequencing and analysis of downstream data have not yet been performed, these findings suggest that KINFix‐fixed samples could be compatible with scRNA‐seq, providing experimental flexibility for studies on rare, field‐collected, or developmentally specific stages of non‐model organisms.

## 5 Conclusion

Our results show that KINFix provides a valuable and cost‐effective fixative for studies with limited biological material that might require different fixation techniques for multiple downstream applications. This is particularly advantageous when working with non‐model organisms, as the material obtained in the field is often limited, and keeping organisms alive or using different fixatives for multiple analyses may not be feasible. Unlike more expensive options such as RNAlater, KINFix provides a versatile, safe, and cost‐efficient solution effective for high‐quality preservation for both morphological and molecular analyses, offering a practical and unique solution for integrative research.

## Author Contributions

**Irene del Olmo:** data curation (equal), formal analysis (equal), investigation (equal), methodology (equal), visualization (equal), writing – original draft (equal). **Paula Moreno‐Martín:** data curation (equal), formal analysis (equal), investigation (equal), methodology (equal), visualization (equal), writing – original draft (equal). **Patricia Álvarez‐Campos:** conceptualization (equal), funding acquisition (equal), resources (equal), supervision (equal), writing – review and editing (equal). **Aida Verdes:** conceptualization (equal), funding acquisition (equal), project administration (equal), resources (equal), supervision (equal), writing – review and editing (equal).

## Funding

This work was supported by the NextGenerationEU (IJC2020‐045256‐I and CNS2023‐145193); 'la Caixa' Foundation (100010434, LCF/BQ/PR24/12050011); Universidad Autónoma de Madrid (SI1/PJI/2019‐00532); and Ministerio de Ciencia e Innovación (MCIN/AEI/10.13039/501100011033).

## Conflicts of Interest

The authors declare no conflicts of interest.

## Supporting information


**Figure S1:** Comparison of ACME‐dissociated cells in live and KINFix‐fixed *Pristina leidyi* individuals.

**Table S1:** Nanodrop and TapeStation measures of all samples used in the study.

## Data Availability

All the required data are uploaded as Supporting Information S1.
