# Supplementary figures and images for: A Field‐Friendly, Non‐Toxic Fixative for Integrated Morphological and Molecular Research in Non‐Model Invertebrates
Source: Ecol Evol. 2026 Feb 10;16(2):e73006. doi: 10.1002/ece3.73006 (PMC12889569; doi:10.1002/ece3.73006)

**(a)** ACME in living organisms

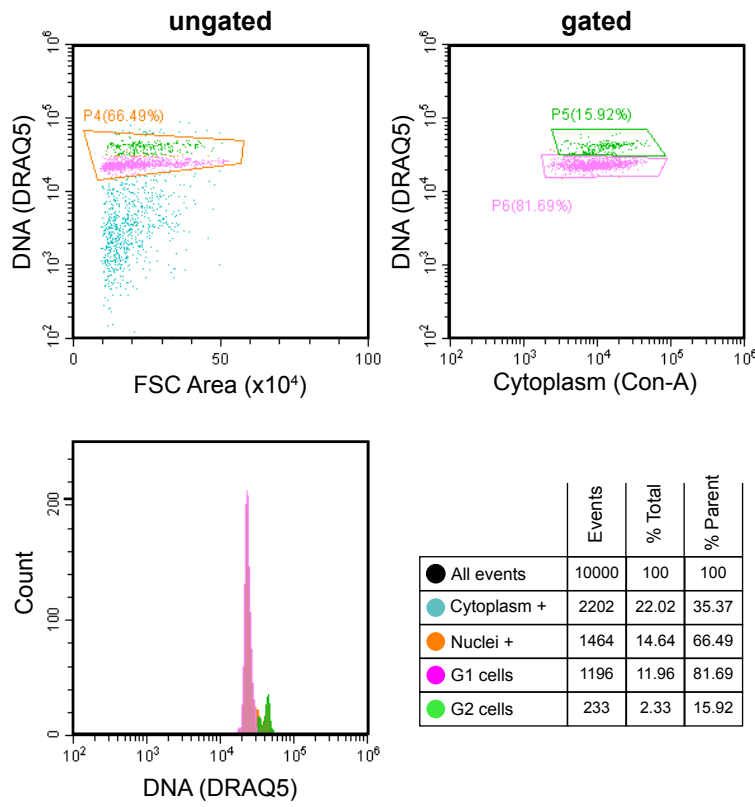

**(b)** ACME in KINFix-fixed organisms

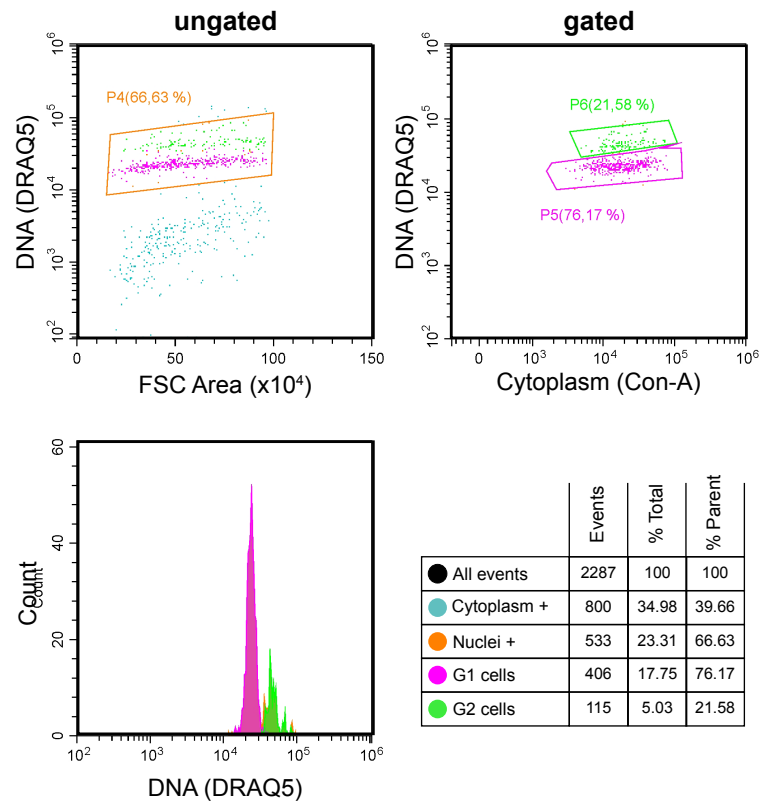

Supplement: Supplementary file 1 — Figure S1: Comparison of ACME‐dissociated cells in live and KINFix‐fixed Pristina leidyi individuals. [file ECE3-16-e73006-s002.pdf]
